# Exosomal Chaperones and miRNAs in Gliomagenesis: State-of-Art and Theranostics Perspectives

**DOI:** 10.3390/ijms19092626

**Published:** 2018-09-05

**Authors:** Celeste Caruso Bavisotto, Francesca Graziano, Francesca Rappa, Antonella Marino Gammazza, Mariantonia Logozzi, Stefano Fais, Rosario Maugeri, Fabio Bucchieri, Everly Conway de Macario, Alberto J. L. Macario, Francesco Cappello, Domenico G. Iacopino, Claudia Campanella

**Affiliations:** 1 Department of Experimental Biomedicine and Clinical Neuroscience, Section of Human Anatomy, University of Palermo, 90127 Palermo, Italy; celestebavisotto@gmail.com (C.C.B.); francyrappa@hotmail.com (F.R.); antonella.marino@hotmail.it (A.M.G.); fabiobuk@hotmail.com (F.B.); francapp@hotmail.com (F.C.); 2 Euro-Mediterranean Institute of Science and Technology (IEMEST), 90136 Palermo, Italy; ajlmacario@som.umaryland.edu; 3 Institute of Biophysics, National Research Council, 90143 Palermo, Italy; 4 Department of Experimental Biomedicine and Clinical Neuroscience, Section of Neurosurgery, University of Palermo, 90127 Palermo, Italy; francesca.graziano03@unipa.it (F.G.); rosario.maugeri1977@gmail.com (R.M.); gerardo.iacopino@gmail.com (D.G.I.); 5 Department of Oncology and Molecular Medicine, National Institute of Health, Viale Regina Elena 299, 00161 Rome, Italy; mariantonia.logozzi@iss.it (M.L.); stefano.fais@iss.it (S.F.); 6 Department of Microbiology and Immunology, School of Medicine, University of Maryland at Baltimore-Institute of Marine and Environmental Technology (IMET), Baltimore, MD 21202, USA; econwaydemacario@som.umaryland.edu

**Keywords:** gliomas, molecular chaperones, Hsps (Heat shock proteins), Hsp60, miRNA, exosomes, extracellular vesicles, theranostic tools

## Abstract

Gliomas have poor prognosis no matter the treatment applied, remaining an unmet clinical need. As background for a substantial change in this situation, this review will focus on the following points: (i) the steady progress in establishing the role of molecular chaperones in carcinogenesis; (ii) the recent advances in the knowledge of miRNAs in regulating gene expression, including genes involved in carcinogenesis and genes encoding chaperones; and (iii) the findings about exosomes and their cargo released by tumor cells. We would like to trigger a discussion about the involvement of exosomal chaperones and miRNAs in gliomagenesis. Chaperones may be either targets for therapy, due to their tumor-promoting activity, or therapeutic agents, due to their antitumor growth activity. Thus, chaperones may well represent a Janus-faced approach against tumors. This review focuses on extracellular chaperones as part of exosomes’ cargo, because of their potential as a new tool for the diagnosis and management of gliomas. Moreover, since exosomes transport chaperones and miRNAs (the latter possibly related to chaperone gene expression in the recipient cell), and probably deliver their cargo in the recipient cells, a new area of investigation is now open, which is bound to generate significant advances in the understanding and treatment of gliomas.

## 1. Introduction

The effective treatment of brain tumors, including gliomas, sadly remains an unmet clinical need. The prognosis is poor even after surgical resection followed by post-operatory chemo- and radiotherapies [1]. It is, therefore, cogent to find groundbreaking treatments. Three recent developments may provide an innovative point of view regarding brain malignancies, opening a novel vision for development of treatment strategies. These new advances are (i) the discovery that molecular chaperones can be determinant factors in the process of tumorigenesis; (ii) the elucidation of the role of miRNAs in gene regulation and determination of protein quantities, including molecular chaperones, in the various cell compartments; and (iii) the increasing understanding and characterization of exosomes, particularly in what refers to their release by tumor cells, contents including chaperones and miRNA, and ability to travel and interact with target cells near their origin or far. This new approach is supported by three main pillars: chaperones, Hsp60-related miRNAs, and exosomes, specifically those released by brain gliomas and distributed over the whole body.

The purpose of this article is to take advantage of these three major scientific advances by calling attention to them, with the purpose of stimulating the investigation in this field and opening our minds with regard to this new platform for studying and managing gliomas. 

As an attractive challenge for the future, we evaluate the possibility to elucidate the role of extracellular chaperones or related miRNAs in brain tumors, to use them as “theranostic tools”. The complete elucidation of these factors as cancer biomarkers has potential advantages, since it should allow the standardization of quantitative tests, with high sensitivity and relatively easy application [2,3,4].

## 2. Gliomagenesis Overview and Clinical and Histopathological Classifications of Gliomas

Glial cells, or neuroglia, are a heterogeneous cellular compartment of the nervous tissue associated with neurons and have various roles in the central and peripheral nervous systems (CNS and PNS, respectively). These cells are more numerous than neurons and are functionally distinct from them, since they are involved in the maintenance of neuronal homeostatic balance and myelination, providing structural support and protection for neurons. Furthermore, during embryonic development, glial cells regulate the differentiation and neuronal survival. In the CNS, glial cells are variable in number and in type, including two main groups: macroglia and microglia. The first group includes the larger types of glial cells originating in the neural plate represented by astrocytes and oligodendroglia, whereas the second group includes smaller types of cells originating in the mesoderm. In the PNS, glial cells are represented by (i) the Schwann cells that develop from the neural crest cells that migrate away from the neural tube; and (ii) satellite cells that appear postnatally.

Until the discovery of neural stem cells (NSCs) in the CNS [5], it was assumed that glial cells were the only ones endowed with the capacity to divide and, consequently, the histological classification of brain tumors was based on glial cells features. In fact, gliomas display histological similarities to glial cells, including astrocytes and oligodendrocytes and, therefore, they are classified as astrocytoma, oligodendroglioma, or oligoastrocytoma. More than half of gliomas are glioblastoma multiforme (GBM; World Health Organization, WHO grade IV astrocytoma) [6,7].

Gliomas and other neuroepithelial tumors constitute 49% of primary brain tumors, while meningiomas are the next most frequent histologic type (27%). Brain metastases affect 9% to 17% of cancer patients; this frequency is proportionate to the relative cardiac output to the brain. 

The worldwide incidence rate is of 14:100,000 person-years for all gliomas [8]. In the United States, it was estimated to be 3:100,000, with more than 10,000 cases being diagnosed annually. GBM constitutes 45.2% of all malignant CNS tumors, 80% of all primary malignant CNS tumors, and approximately 54.4% of all malignant gliomas. Mean age at diagnosis is 64 years, and it is 1.5 times more common in men than in women and 2 times more common in whites compared to blacks [1]. The incidence has increased slightly over the past 20 years, mostly due to improved radiologic diagnosis and increase in life span of men and women [9]. 

Recent genetic studies have identified a number of recurrent chromosomal abnormalities and genetic alterations in malignant gliomas, particularly in GBM [10]. 

The main biological processes in gliomagenesis are extracellular matrix (ECM) remodellng and epithelial–mesenchymal transition (EMT). Tumor cell growth and invasion are linked to ECM remodeling, involving proteolysis. Physiologically, the basement membrane (BM) separates epithelial cells from the ECM preventing their interaction with the microenvironment. Destabilized BM allows contact between epithelial cells and various signaling ECM proteins [11]. Expression of mesenchymal cytoskeletal proteins and deposition of ECM proteins promote the migratory potential of glioma cells by activating integrin and signaling pathways. These changes activate the invasiveness and migratory potential of glioma cells, leading to poor prognosis with evasion from treatment agents and establishment of resistance to therapeutics [12].

One of the major challenges in glioma treatment is the migratory potential of glioma cells breaching into the blood–brain barrier (BBB)-protected areas of the brain. Hence, unravelling the migration mechanisms has always been of great importance in glioma research [13].

The major players contributing to glioma cell dissemination are ECM degradation through matrix metalloproteases (MMPs), intracellular cytoskeletal rearrangements, and stimuli by chemoattractants. The MMPs mainly aid in degrading the basement membrane along with ECM. During EMT, MMPs secreted by glioma and stromal cells facilitate ECM remodeling and invasiveness through ECM degradation, promoting migration due to release of growth factors embedded within the ECM for activation of signal transduction cascades. The levels of MMPs are correlated with histological grading of gliomas [13].

Clinically, patients with GBM may present with headaches, focal neurologic deficits, confusion, memory loss, personality changes, and seizures. Diagnosis and response to treatment are and monitored by magnetic resonance imaging (MRI) and the use of adjunct technology, such as functional MRI, diffusion-weighted imaging (DWI), diffusion tensor imaging (DTI), dynamic contrast-enhanced MRI, perfusion imaging, magnetic resonance spectroscopy (MRS), and positron-emission tomography (PET) [14].

Currently, maximum safe optimal surgical resection, followed by partial brain radiotherapy with concurrent temozolomide, and subsequent continuation of temozolomide for 6 cycles, is considered the standard treatment modality. However, the overall survival for GBM patients is only around 9–12 months, and the overall 5-year survival rate is less than 5%. In this context, the future direction in GBM management should include the “molecular approach”, in order to achieve strictly pertinent selection of patients and make the therapeutic protocols personalized and more appropriate.

## 3. Molecular Chaperones: Locales of Residence, Functions, and Roles during Tumorigenesis

Molecular chaperones, many of which are heat shock proteins (Hsps), are an important class of molecules with various functions, intra- and extracellularly. Not all Hsps are chaperones, but the terms chaperone and Hsp are used interchangeably, even if strictly they are not synonyms. 

These molecules, for the most part conserved during evolution, are involved in the maintenance of protein homeostasis by assisting in the folding of client polypeptides, refolding of partially denatured proteins, and degradation of proteins damaged beyond repair (references in [15]). Chaperones also participate in protein translocation across membranes. One might say that chaperonology is a scientific discipline that studies molecular chaperones and the pathological conditions in which chaperones may become pathological factors, known as chaperonopathies [16]. Chaperone therapy, or chaperonotherapy, involves the use of chaperones in the treatment of chaperonopathies [17,18]. A practically useful classification of chaperones groups them by molecular weight as follows: super heavy, 100, 90, 70, 60, 40, small Hsp (sHsp), including chaperones within the following ranges in kDa: 100 or higher, 81–99, 65–80, 55–64, 35–54, and 34 or lower, respectively [19,20].

The Hsps were initially described as a group of intracellular proteins whose genes are induced by heat shock, as well as by other stressors [21]. Expression of Hsps occurs in response to a wide variety of stressors, such as hyperthermia, hypoxia, ischemia, heavy metal or ethanol exposure, and infections [13]. Most Hsp chaperones are involved in various physiological mechanisms in normal cells, such as DNA replication and gene expression regulation [22,23]. Furthermore, molecular chaperones have other roles, such as participation in immune system regulation [18,24], cell differentiation [25] and apoptosis, and carcinogenesis, including GBM [15,26]. The levels of some Hsps are elevated in various types of cancer, which usually indicates poor prognosis in terms of survival and response to therapy [21]. Numerous studies have shown that Hsps are involved in malignant transformation, metastasization, and appearance of multidrug-resistance [26,27]. In addition, elevated levels of intracellular Hsps can protect malignant cells against therapy-induced apoptosis [22,26].

Chaperones have canonical and non-canonical (moonlighting) functions, with the former pertaining to the maintenance of protein homeostasis and the latter pertaining to other aspects of cellular and organismal physiology unrelated to protein homeostasis. Chaperones are components of the chaperoning system (CS) of an organism together with co-chaperones, chaperone co-factors, and chaperone receptors and interactors [19,20]. The main partners of the chaperoning system in the maintenance of protein homeostasis (i.e., the canonical functions) are the ubiquitin–proteasome system (UPS) and the chaperone-mediated autophagy (CMA) machinery, whereas the major partner for non-canonical functions is the immune system (IS). When the interaction between the CS and the IS is altered (e.g., chaperone mutation, or overexpression, or post-translational modification) chronic inflammatory and autoimmune disorders, and cancer progression and metastasization, may occur. 

## 4. miRNAs: Intracellular Localization, Functions, and Roles during Tumorigenesis

The realization that tumorigenic mechanisms develop because of alterations at various levels of the gene expression regulation, including chaperone genes, opened new avenues to study the role of non-coding nucleic acid molecules in cancer.

There is a large number of non-coding (i.e., they do not encode proteins) nucleic acids which have different structures, and which act as key regulators of gene expression in many different cellular pathways and locales. About 98–99% of the human genome encodes molecules considered for a long time as “dark matter” whose biological significance has long been debated. Nevertheless, in the last twenty years, non-coding RNA (nc-RNA) has been widely studied, and its complex role in pathophysiological mechanisms is now better understood [28,29,30,31].

The term “non-coding RNAs” describes a large group of RNAs that contains subgroups with different functions. They are classified based on the molecular length, as follows: non-coding RNAs that have more than 200 nucleotides in length are “long” non-coding RNAs (lncRNA), while those that have less than 200 nucleotides are the “small” non-coding RNAs (sncRNAs). lncRNAs appear to be epigenetic regulators affecting, at various levels, protein-coding gene expression. sncRNAs seem to be involved in many, if not all key cellular activities, including development and differentiation, and transcriptional and post-transcriptional gene silencing, all functions that seem to be determined by the subcellular localization [32]. sncRNAs include various different small RNAs, such as microRNAs (miRNAs), small nucleolar RNAs (snoRNAs), and piwi-interacting RNAs (piRNAs) [33]. miRNAs recognize target RNAs and play a key role in modulating gene expression in a sequence-specific manner. A number of studies on miRNA expression profiles and on inhibition of pathogenic miRNAs have been at the basis of efforts for developing new tools for diagnosis and assessing prognosis and response to treatment and also novel therapeutic agents and strategies [34,35]. 

Most microRNAs are transcribed by RNA polymerase II under the control of various transcription factors, as long transcripts characterized by a complex hairpin structure (pri-miRNA), and are processed in the nucleus by a RNase III, named Drosha, to generate the microRNAs precursors (pre-miRNA) with a length of 70–100 nucleotides. These precursors are transferred from the nucleus to the cytoplasm, in which they undergo further maturation mediated by another RNase III (Dicer), generating a double-stranded RNA (dsRNA) of 22 nucleotides. This dsRNA is subsequently incorporated in a protein complex, known as RISC (RNA-induced silencing complex), which promotes the formation of single-stranded mature molecules. In the RISC protein complex, mature miRNAs are able to pair with complementary sequences present in the 3′-UTR region of mRNAs and to regulate gene expression at the post-transcriptional level. miRNAs may induce mRNA degradation or blockage of translation without mRNA degradation, depending on the complementarity of the bases between the miRNA and its target site [36]. 

The finding that the miRNA miR-15a and miR-16a gene sequences were deleted or downregulated in the majority of B-cell chronic lymphocytic leukaemias (B-CLL) [37], was one of the first indications of the involvement of miRNAs in human tumorigenesis. As a consequence, many studies were carried out on the correlation between genomic position of miRNA genes and the cancer-associated genomic regions, accompanied by miRNAs overexpression/downregulation that resulted in transcriptional control and epigenetic changes and in defects in the miRNA biogenesis machinery [38,39,40].

The mechanism of miRNAs dysregulation in cancer involves amplification or deletion of their genes [37,39], and abnormal expression of transcriptional factors that regulate miRNAs expression [41]. Other factors that generate miRNAs dysregulation leading to tumorigenesis are epigenetic alterations, i.e., aberrant DNA methylation and histone acetylation of miRNAs genes [42], and defects in enzymes involved in the miRNAs maturation steps [43].

The identification of different expression profiles of miRNAs in the neoplastic tissue compared with its normal counterpart supports the hypothesis of a probable involvement of miRNAs in tumor development and progression, including evasion of apoptosis, and also induction of cell migration, epithelial–mesenchymal transition (EMT), and angiogenesis [44,45]. The expression of miRNAs, in different tumor-cell types (lung, breast, stomach, prostate, colon, and pancreas), was found distinctive of tumor type, suggesting a definite role of miRNA in human tumorigenesis and indicating that miRNA expression is a signature of the tumor cell that could be used for tumor classification, diagnosis, and assessing prognosis [46].

Little is known about the cellular sites where miRNA-mediated post-transcriptional regulation occurs. It has been shown that Argonaute (AGO) protein–miRNA complex is present in cellular organelles, including endoplasmic reticulum (ER), Golgi apparatus, lysosomes, and endosomes [47,48,49]. miRNAs have been found in different cellular fractions, and in the extracellular environment, into which they would be secreted by passive and/or active mechanisms, for example, by loading them onto extracellular vesicles, such as exosomes [50]. In the passive mechanism, miRNA secretion would be driven by the natural affinity of the nucleotide sequences with lipid rafts [51], whereas the active mechanisms (regulated secretion) would be due to translocation into microvesicles that act as signal transporters and mediate long-range cell–cell communication [52].

Numerous studies have shown the presence of miRNAs within the exosomes, and that the variation of the intracellular amounts of individual miRNAs was reflected within the exosomes [53]. It could very well be that miRNAs released with the exosomes from cancer cells are able to alter gene expression in surrounding and far away tissues, and thereby contribute to the progression of tumors [54]. Currently, the mechanism of miRNA sorting into the exosomes is still poorly understood.

## 5. Exosomes: Nanovectors for Extracellular Chaperones and miRNAs

Exosomes are extracellular nanovesicles of 20–100 nm in diameter, of endosomal origin, that are secreted by virtually all cell types, probably to exchange information and to maintain cellular homeostasis, for example, in response to pathogens. Exosomes play an important role in cell–cell communication, different from that occurring by the classical pathway involving direct cell–cell contact and secretion of soluble factors [55,56].

It was initially thought that exosomes could be part of a mechanism for shedding the cytoplasm content during maturation of reticulocytes [57]. Several studies provided further support to show that exosomes are also present in body fluids, such as blood, urine, breast milk, saliva, and bronchoalveolar lavage, and cerebrospinal, ascitic, and amniotic fluids [56]. Clinical trials have shown that exosomes can be characterized and quantified in the plasma of healthy subjects and of patients with tumors of different histology [4,58], using different techniques, including immunocapture-based Enzyme Linked Immunosorbent Assay (ELISA), Nanoparticle tracking analysis (NTA), and nanoscale flow cytometry. This approach allowed to compare old and new disease biomarkers and also to quantify the plasmatic levels of exosomes, providing new insights on this issue. This was an important contribution, since chronic disease conditions may be characterized by higher plasmatic exosome levels, as compared to both healthy and other diseased controls [4,58], suggesting that, at least in tumor patients, higher plasmatic levels of exosomes may represent a marker of disease, per se [59].

Exosomes are released into the extracellular space after the merging of late endosomes with the cell membrane. This release is preceded by early endosomes becoming part of multivesicular bodies (MVBs), which undergo a maturation process that determines a gradual change in molecular composition of the vesicles (intraluminal vesicles, ILVs). The vesicles in the MVBs merge with the lysosomes during this maturation process, which causes degradation of their contents (e.g., in the case of receptors), or constitutes a temporary storage compartment, or they may blend with the plasma membrane, releasing exosomes. In brief, MVBs merge with the plasma membrane, resulting in exocytosis of the vesicles contained in MVBs, i.e., exosomes [58,59].

Exosomes have cell type-specific contents. Numerous studies are being conducted to elucidate the contents of exosomes with the purpose of determining their functions and, ultimately, use them in therapeutics, for example, as vehicles for drug or chaperone delivery [60,61]. Various functions have been attributed to exosomes, depending on the source cell and their contents. For instance, exosomes are involved in cell-to-cell information transfer [62], inflammation [63], coagulation [64], stem cell activation [65], programmed cell death [66], and the immune response [63].

It seems plausible that the exosomes’ composition depends on the parental cells contents, including proteins; lipids; nucleic acids, such as DNA and non-coding RNA (e.g., ribosomal RNA (rRNA), and miRNAs).

Different sets of proteins are found in exosomes, some strictly involved in vesicle trafficking, such as cell surface receptors; others pertaining to the endocytic pathway (i.e., endosomal sorting complex required for transport, ESCRT, such as Alix; tumor susceptibility gene 101, TSG101; integrin; and tetraspanins); and still other proteins involved in long distance communication, such as cytokines [67], hormones [68], growth and transcription factors [69], and Hsps [68,69].

Hsp60, Hsp70, and Hsp90 are secreted by cancerous cells via the exosome pathway [63,64,65]. Exosomal Hsps may have opposite effects: immunosuppressing or immunostimulating. These different effects depend on the interaction between exosomal Hsps and cells of the immune system. For instance, Hsp60, Hsp70, and Hsp90α are actively secreted via the exosomal pathway, and mediate immunomodulatory effects and immune response against cancer cells [70,71]. Extracellular Hsp70 activates macrophages [72,73,74] and natural killer cells [75,76], while Hsp90α, when released by invasive cancer cells via exosomes, enhances cancer cell migration [77]. 

Our research group has provided one of the first pieces of evidence that Hsp60 is present in exosomes, including the mechanism by which the chaperonin is loaded onto exosomes, and its location in them [70,78]. Under normal conditions, Hsp60 acts as a molecular chaperone intracellularly but, on the other hand, in several pathological states it is overexpressed and is released by both the classical pathway and via exosomes. In heart failure, Hsp60 is secreted by cardiomyocytes, and its presence in the serum correlates with disease severity and cardiovascular risk [76,78]. Hsp60 is released by adult cardiomyocytes via the exosome pathway in both the basal state and following mild stress [79]. Fibrosarcoma cells release Hsp60 through the conventional endoplasmic reticulum–Golgi protein transport pathway [80]. In in vivo studies, we showed that the exosomal Hsp60 levels in the plasma of patients before colon cancer surgery were significantly higher than in the exosomes from the same patients after tumor ablation [71]. Hsp60 exportation by exosomes, which would undergo post-translational modifications [81], points to roles of this chaperonin in inflammation, immune system modulation, and regulation of tumor microenvironment and growth. Therefore, exosomal Hsp60 may contribute to the regulation of gene expression in target cells at distant sites (reviewed in [2]).

The presence of mRNA [82] and miRNA [83] in exosomes suggest regulatory activity on gene expression in recipient and donor cells, and horizontal transfer of genetic information.

The mechanism by which miRNAs are loaded on to exosomes remains unclear. It is debated whether miRNA molecules are specifically charged onto exosomes, or incorporated passively, as a result of mutual affinity between repeated RNA sequences and lipid rafts. As is known, during maturation, the miRNA–protein complex (miRISC) is incorporated in late endosomes and subsequently in exosomes, but certain exosomal miRNAs are independent of miRISC [47]. On the other hand, specific sequences present in certain miRNAs may guide their incorporation into exosomes [84], a possibility supported by the finding that exosomes from diverse sources, including several human body fluids, contain only one copy of a given abundant miRNA, because many more copies would be expected from random sampling. This suggests a tightly regulated sorting mechanism that has yet to be elucidated. The number of molecules that are loaded within exosomes and whether it can actually determine a modulation of gene expression in the recipient cells are still under scrutiny. In conclusion, it has been shown by various researchers [84,85,86,87] that specific mechanisms for miRNAs sorting into secreted vesicles do exist, and which molecules are actually sorted depends on the translational status in the source cells. Therefore, one can speculate that specific miRNAs encapsulated in exosomes can educate the recipient cells, a possibility that, if proven, will represent an important pathophysiologic event in long distance cell–cell communication. In this regard, it has to be emphasized that miRNAs are preserved in a stable form inside the exosome, in which they are protected from endogenous RNase activity.

## 6. Chaperones, miRNAs, and Exosomes as Novel Molecular Target to Be Explored for Diagnosis and Prognosis Assessment of Gliomas

Since the main biological processes in gliomagenesis are extracellular matrix (ECM) remodeling and epithelial–mesenchymal transition (EMT), efforts have been directed to elucidate the molecular mechanisms involved. The dysregulation of the activity of glioma cells can induce in them the capacity to release different class of molecules, with effect on the surrounding environment. This release may be mediated by exosomes, which induce modifications of the recipient cell phenotype and remodeling of ECM, causing cancer propagation [88]. 

**Figure 1:**
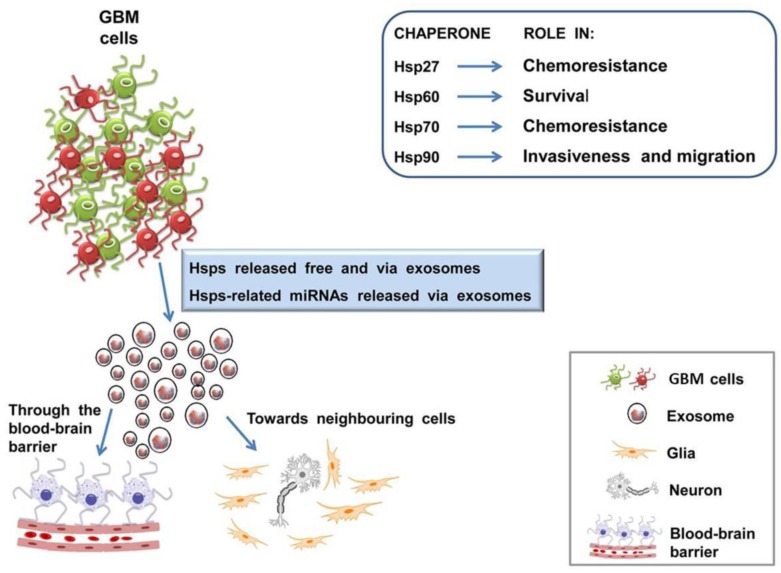
Chaperones and exosomes from glioblastoma multiforme (GBM) cells are central and conspicuous players in patients and constitute attractive targets for therapeutics along with pertinent miRNAs. The figure presents the elements that provide the foundations for research toward the development of new therapies and summarizes the main thesis of this review, as a proposal for future research, since various points are still under scrutiny. GBM cells differentially express various Hsps, which play pivotal roles in chemoresistance, apoptosis escape, invasiveness, and cell migration (shown in the top right inset). Hsps also occur extracellularly, free or in exosomes released by GBM cells carrying these proteins and miRNAs. Exosomes with their cargo would participate in the modulation of the immune system and in the regulation of gene expression in the target cells, and would modify the tumor microenvironment, ultimately favoring tumor dissemination, for instance, through the blood–brain barrier. The challenge consists in manipulating these elements to use them as therapeutic agents. Icons are explained in the bottom right inset.

It is well established that brain-derived exosomes can cross the blood–brain barrier (BBB) [89,90,91,92], probably by a mechanism involving their surface epitopes [93,94], and so they can be found in plasma. Tumor cells produces exosomes in markedly altered numbers and composition, although nowadays, their functions in circulation still remains unclear, but it is believed that they influence tumor growth and progression [59]. Therefore, in normal and pathological conditions, the nervous cells release, via the exosomal pathway, several bioactive molecules, participating in long-distance communication (Figure 1). The analysis of exosome contents could be a possible way for monitoring the changes occurring in brain, offering new promising approach for biomarkers discovery and for identifying novel targets for treatment.

Extracellular vesicles isolated from GBM cells carry a variety of proteins, nucleic acids, lipids, and other metabolites, that represent accessible biomarkers for diagnosis and for assessing response to treatment response [95]. The proteins are involved in pathways for remodeling of ECM, biological adhesion, developmental processes, and progression of inflammation, leading to cancer growth [95]. Among the proteins that were detected in exosomes produced from glioma cells, we can mention the coatomer protein complex subunit; collagen type VI; myosin heavy chain 1; keratin; annexin A1, A2, A4, A5, A6, A11; Ras-related protein 10 (Rab10), Rab7a, Rab5c; type I collagen and type VI alpha 1 collagen; integrins (β1, α3, αV); CD44; and different kinds of tubulins and actinins [96,97]. The surface of exosomes isolated from GBM cells present the specific mutant epidermal growth factor receptor variant III (EGFRvIII), which is the best known and most specific protein marker for GBM [98]. Although evaluation of EGFRvIII protein levels would be the most direct assessment, availability of antibody reagents is limited, resulting in the use of nucleic acid techniques for detection [99]. Other studies identified MMP9, TGF(Transforming growth factor)-β, IL(interleukin)-10, and isocitrate dehydrogenases 1 (IDH1) and 2 (IDH2) as components of extracellular vesicles and exosomes cargo produced by GBM cells or isolated from blood [98]. These proteins have not been proposed as specific markers, since they were found in both cancer and normal cells. Still other proteins, such as Hsps (Hsp27, Hsp60, Hsp70, Hsp90, Hsp110) have been found [97].

In glioblastoma, as well as in other cancers, Hsps display chaperoning activity to assist various components of the ECM remodeling machinery, and a positive correlation has emerged between high expression of Hsps and the triggering of the invasive and metastatic potentials of glioma cells [100]. Poor prognosis and resistance to therapeutics are closely associated with elevated expression of Hsps in gliomas. Hsps promote cancer growth by stimulating cell proliferation and inhibiting cell death pathways [101,102]. In glioma cell lines, Hsp27, Hsp70, and Hsp90 play key roles in pathways involved in survival, invasiveness, and migration [103,104,105,106,107] (Table 1). Furthermore, Hsp60 promotes glial cell proliferation with the chaperonin being differentially expressed in glioma tissue, which contrasts with surrounding normal areas [108]. It is likely that extracellular Hsps participate in immune system stimulation or, on the other hand, they may favor tumor escaping from immune reaction. Hsp participation in cell-to-cell crosstalk [74,96] is made possible by their being on the exosome’s surface, and being secreted by both normal and tumor cells [109]. Hence, Hsps may be important for intercellular cross talk, and when present on the exosome surface, can enhance the activity of the immune system [2,71,72,110]. Thus, exosomes containing Hsps have high potential for both diagnostic and therapeutic applications, and may be considered candidates, alone or in combination with other biomarkers, for use as potential antitumor vaccines or immunotherapeutic vehicles for targeting malignant cells. Of note, the determination of exosomal Hsp is relatively easy by high throughput proteomic analysis or by a simple Western blot, involving a non-invasive method such as the withdrawal of a blood sample. 

Brain tumor tissue studied by Western blot and immunohistochemistry (IHC) showed a marked increase in the constitutive expression of Hsp27, Hsp90, GRP (glucose regulated proteins) 78 and 75, and Hsc70 (or Hsp70) and the co-chaperone HspBP1 (Hsp70 Binding Protein 1) [112,114,115] (Table 1). Experimental in vivo tumors grown from glioma cell lines express Hsp27 and Hsp70 at high levels [103,116]. Hsp27, Hsp60, Hsp70, and Hsp90, namely the Hsps, overexpressed in glioma cells, can all be released via exosomes [112] (Table 1).

These observations, together with the finding that anticancer agents can cause resistance to chemo- and radiotherapies [117], encourage us to consider Hsps as targets of an effective strategy for management of human gliomas.

The role of Hsp60 on gliomagenesis is still poorly understood, although it has been established that it is differentially expressed in glioblastoma cell lines [118,119] (Table 1). Depletion of Hsp60 in in vivo models of GBM led to suppression of the intracranial tumor [118]. 

Hsp60 upregulates various pathways that provide survival benefits to malignant cells, bypassing apoptosis and/or senescence [81], and probably, the chaperonin regulates the miRNAs pathway, too. Increase or decrease in the levels of Hsp60 and the pathophysiological significance of these quantitative variations seem to be dependent on the locale in which the chaperonin resides [120]. Cytosolic Hsp60 has a pro-apoptotic role: it binds pro-caspase 3 allowing its activation in normal cells [121] but, in tumor cells, Hsp60 does not activate the caspase pathway assuring malignant cell survival and, thus, contributes to tumorigenesis [122] by playing an antiapoptotic role [123]. It has been observed that extracellular Hsp60 can mediate apoptosis via its ligand (e.g., Toll-like receptor 4), causing a pro-survival effect in glial cells [113]. Hsp60 is also involved in microglial activation, leading to overproliferation under abnormal conditions [124] (Table 1). It has been reported that the presence of Hsp60 in the surface of astrocytes, which are known to interact continuously with the microglia in both physiological and pathological conditions, could have a role in the protection of the brain [124]. Surprisingly, this hypothesis appears to be in contrast to the pro-inflammatory role of extracellular Hsp60, but now it is widely accepted that Hsp60 has multifaceted roles, for instance, depending on its tissue localization [71]. Moreover, it has been shown that Hsp60 is released by cells, likely through exosomes, possibly coordinating various functions of the brain cell types [124]. 

Besides the multiple roles in cell homeostasis regulation, Hsps participate in several tumorigenic pathways, such as the inhibition of caspase-dependent pathways [125], or through favoring the cell morphology modification, migration and invasion [103,126], and inducing neoangiogenesis [127]. On the other hand, Hsp40 has been implicated in contrary effects, because it also induces apoptosis [128]. However the underlying mechanisms are not fully elucidated and, until now, only few inhibitors have been developed against several Hsps, reporting promising results, but at the initial stage, in other cancer types [129,130,131,132]. Further studies of molecular characterization of Hsps would be needed in order to use these proteins as therapeutic targets in GBM. It has been observed that the functional significance of Hsps seems to be dependent on their localization, and it is possible that glioma cells use exosomes to release Hsps [124] as a means to modulate the immune system and, probably, to release miRNAs molecules, targeting Hsp gene sequences, for example (Figure 1). This would result in a modulation of gene expression in the target cells, determining a modification of the tumor microenvironment and favoring tumor dissemination. 

Regarding the regulatory potential of miRNAs in Hsp expression, it has been demonstrated that miR-628-3p affected migration and apoptosis in lung cancer cells, downregulating Hsp90a protein expression via a post-transcriptional mechanism [133], and that the oncogene Hsp90B1 is a direct target of the tumor suppressor miR-223 in a cell model of human osteosarcoma [134]. Likewise, it has been established that hyperthermia-induced overexpression of Hsp90 was suppressed by miR-27a in human oral squamous cell carcinoma HSC-4 cells [135]. MiR-142-3p has been indicated as a novel regulator of Hsp70 expression in triptolide-induced pancreatic cancer cell death [136], and it has been reported that miR-1/miR-206 suppressed Hsp60 expression, contributing to glucose-mediated apoptosis via the MEK(extracellular signal-regulated kinase)-1/-2 pathway in cardiomyocytes [137]. Also, miR-382 contributes to renal tubule-interstitial fibrosis by downregulating Hsp60 expression [138]. Inhibitors of miR-29a promote apoptosis through upregulation of Hsp60 level and downregulation of Hsp27, Hsp40, Hsp70, and Hsp90 levels in breast carcinoma MCF-7 cells, suggesting a possible alternative for current chemotherapy with fewer side effects [139]. These data suggest that cancer cells use Hsps as an escape mechanism from apoptosis, therefore, their inhibition by modulating miRNAs may provide a novel antitumor therapy, particularly for gliomas. 

## 7. Potential Therapeutic Applications and Conclusions

The conventional treatment strategy for glioma entails maximal surgical abscission, radiotherapy, and concomitant/adjuvant chemotherapy [140,141]. Despite contemporary treatments, the prognosis for GBM patients remains dismal. A number of difficulties confront the physician. For instance, some brain lesions are not surgically accessible, are genetically and phenotypically heterogeneous, and may possess acquired resistance to therapeutics because GBM cells are endowed with various active key oncogenic pathways and effective cytoprotective mechanisms. Over the last years, research efforts have been aimed to discover genetic alterations or aberrant signaling pathways, in order to understand the biology of gliomas. Genomic, transcriptomic, and proteomic profiling may unveil new molecular aspects yet unknown of high-grade gliomas. It is known that molecular chaperones potentially confer resistance against chemo- and radiotherapies [26,27,142,143] and support glial tumor growth and invasion [13]. Given their functions, Hsps are able to interact with a wide spectrum of molecules, playing a cytoprotective role against various types of cellular stress. Under pathological conditions, Hsp dysregulation could favor cell survival in the face of lethal injuries, leading to apoptosis inhibition. This phenomenon would be increased in the tumor environment by molecular effectors, such as miRNAs, which has been shown to have as targets several pathways involved in GBM [144] and have, as downstream targets, some mRNAs for Hsps or for molecules connected to Hsp pathways [135,145,146,147,148]. miRNAs may also be released by tumor cells, through exosomes for instance, determining gene expression regulation with both autocrine and paracrine modalities, and promoting tumor growth in the surrounding tissues. These players constitute a complex molecular signature that, if fully elucidated, represent an important step towards the definition of biological markers for brain malignancy and for developing specific treatments for patients. 

The search for effective glioma patient stratification based on molecular features could be achieved by exploiting chaperones or their regulators as therapeutic targets. Anti-GBM vaccines, specific chaperone inhibition, and drug (natural/synthetic) discovery strategies are some of the most promising novel theranostic additions in the field of glioma therapy.

There is encouraging potential to enhance the research on molecular chaperones that, in the near future, could be proposed as novel biomarkers for diagnosis, assessing prognosis and follow-up of a variety of pathological conditions, or for therapeutic applications (Figure 1).

A major obstacle in glioma treatment is that, on exposure to chemo- and radiotherapies, a number of patients develop resistance to these therapeutic agents associated with a marked increase in Hsps [142,143]. It has also been observed in a number of cases that inhibition of Hsps causes regression of ECM and EMT markers with an increase in cell sensitivity to chemo- and radiotherapies [13]. One may then infer that molecular chaperone Hsps are involved in conferring chemo/radioresistance to glioma patients, thus acting as cytoprotectants for cancer cells. It follows that the therapies that act in conjunction with chaperone inhibitors, negative chaperonotherapy [22], may prove to be an effective strategy in glioma treatment.

Various novel strategies are being used or are good prospects, such as Hsp vaccines, Hsp inhibitors in combination with anticancer drugs, Hsps as vaccines on exosome surface, and miRNA technology, all means with the potential ability to overcome the threat posed by Hsps as cytoprotective of tumor cells. The addition of such novel approaches to conventional treatment strategies and the characterization of the mechanisms involved in the Hsps’ influencing ECM and EMT will certainly aid in glioma management.

It can be concluded that it would be beneficial to look at the CNS tumors through the chaperone eye, so to speak, and consider at least some of these tumors to be chaperonopathies by mistake or collaborationism [17]. In these chaperonopathies, one or more chaperones favor tumor growth, instead of protecting the organism as they are supposed to do. Therefore, looking at tumors through the chaperone eye implies that patient examination should include qualitative and quantitative analyses of Hsps, including those in exosomes, before and after surgery, and other treatments for monitoring disease evolution and response to treatment [149]. This conduct will provide insights on brain tumors that will enhance progress in clinical applications of chaperones and exosomes, including their use as therapeutic agents.

Another promising avenue stems from the fact that cancer-related miRNAs associated with Hsp genes and pseudogenes have been determined for different cancer type as reported above [139]. Similar types of characterization of miRNAs, associated to Hsp genes in GBM, could guide diagnosis, and would aid in the designing of new anti-glioma therapies [121,122].

## Figures and Tables

**Table 1:** Involvement of molecular chaperones in glioma genesis and pathology.

Main GBM-Related Chaperones ^1^	Functions	Ref.
Hsp27	Phosphorylated Hsp27 at high levels co-localizes with secreted protein acidic and rich in cysteine (SPARC), and associates with changes in cell morphology, migration, and invasion in vitro.	[102]
	Hsp27 overexpression is observed in parallel to the increase in malignancy, as a predictive factor of poor prognosis for GBM.	[111]
Hsp60	Hsp60 overexpression inhibit tumor cell death or antitumor immune system response.	[108]
	In glioma cell line, Hsp60 has an antiapoptotic function through CypD-mediated mitochondrial permeability regulation.	[112]
	Hsp60 binds triggering receptor expressed in myeloid cells 2 (TREM2), increasing phagocytic activity in the N9 microglial cell line.	[113]
Hsp70	Hsp70 stabilize the activating transcription factor 5 (ATF5), determining a pro-survival effect in C6 and U87 cells.	[104]
	Hsp70 overexpression inhibits tumor cell death or antitumor immune system response.	[108]
Hsp90	Hsp90 binds and stabilize its client protein (e.g., PTEN, p53, and EGFR) to maintain its expression, leading to aggressive growth in GBM.	[105]
	Hsp90 co-localize with ALDH (aldehyde dehydrogenase) in cancer stem cell sub-population.	[107]
	Extracellular Hsp90α favors cell migration of glioblastoma U87 cells.	[112]

^1^ Abbreviations: GBM, glioblastoma multiforme; Cyp, cytochrome P450; PTEN, phosphatase and tensin homolog (phosphatidylinositol-3,4,5-trisphosphate 3-phosphatase); EGFR, epidermal growth factor receptor.

## Abbreviations

CNS	central nervous system
PNS	peripheral nervous system
NSCs	neural stem cells
GBM	glioblastoma multiforme
WHO	world health organization
ECM	extracellular matrix
EMT	epithelial to mesenchymal transition
MMPs	matrix metalloproteases
DWI	diffusion-weighted imaging
DTI	diffusion tensor imaging
DTT	diffusion tensor tractography
MRI	magnetic resonance imaging
MRS	magnetic resonance spectroscopy
PET	positron emission tomography
Hsp	heat shock protein
MVB	multivesicular bodies
ILVs	intraluminal vesicles

## References

[1] Ostrom Q.T., Gittleman H., Farah P., Ondracek A., Chen Y., Wolinsky Y., Stroup N.E., Kruchko C., Barnholtz-Sloan J.S. (2013). CBTRUS Statistical Report: Primary Brain and Central Nervous System Tumors Diagnosed in the United States in 2006–2010. Neuro. Oncol..

[2] Caruso Bavisotto C., Cappello F., Macario A.J.L., Conway de Macario E., Logozzi M., Fais S., Campanella C. (2017). Exosomal Hsp60: A potentially useful biomarker for diagnosis, assessing prognosis, and monitoring response to treatment. Expert Rev. Mol. Diagn..

[3] Fais S., O’Driscoll L., Borras F.E., Buzas E., Camussi G., Cappello F., Carvalho J., Cordeiro da Silva A., Del Portillo H., El Andaloussi S. (2016). Evidence-Based Clinical Use of Nanoscale Extracellular Vesicles in Nanomedicine. ACS Nano.

[4] Iessi E., Logozzi M., Lugini L., Azzarito T., Federici C., Spugnini E.P., Mizzoni D., Di Raimo R., Angelini D.F., Battistini L. (2017). Acridine Orange/exosomes increase the delivery and the effectiveness of Acridine Orange in human melanoma cells: A new prototype for theranostics of tumors. J. Enzym. Inhib. Med. Chem..

[5] Clarke D.L., Johansson C.B., Wilbertz J., Veress B., Nilsson E., Karlström H., Lendahl U., Frisén J. (2000). Generalized potential of adult neural stem cells. Science.

[6] Crocetti E., Trama A., Stiller C., Caldarella A., Soffietti R., Jaal J., Weber D.C., Ricardi U., Slowinski J., Brandes A. (2012). Epidemiology of glial and non-glial brain tumours in Europe. Eur. J. Cancer.

[7] Alifieris C., Trafalis D.T. (2015). Glioblastoma multiforme: Pathogenesis and treatment. Pharmacol. Ther..

[8] De Robles P., Fiest K.M., Frolkis A.D., Pringsheim T., Atta C., St Germaine-Smith C., Day L., Lam D., Jette N. (2015). The worldwide incidence and prevalence of primary brain tumors: A systematic review and meta-analysis. Neuro-Oncology.

[9] Stupp R., Brada M., van den Bent M.J., Tonn J.C., Pentheroudakis G., ESMO Guidelines Working Group (2014). High-grade glioma: ESMO Clinical Practice Guidelines for diagnosis, treatment and follow-up. Ann. Oncol..

[10] Appin C.L., Brat D.J. (2014). Molecular Genetics of Gliomas. Cancer J..

[11] Iser I.C., Pereira M.B., Lenz G., Wink M.R. (2017). The Epithelial-to-Mesenchymal Transition-Like Process in Glioblastoma: An Updated Systematic Review and In Silico Investigation. Med. Res. Rev..

[12] Carro M.S., Lim W.K., Alvarez M.J., Bollo R.J., Zhao X., Snyder E.Y., Sulman E.P., Anne S.L., Doetsch F., Colman H. (2010). The transcriptional network for mesenchymal transformation of brain tumours. Nature.

[13] Rajesh Y., Biswas A., Mandal M. (2017). Glioma progression through the prism of heat shock protein mediated extracellular matrix remodeling and epithelial to mesenchymal transition. Exp. Cell Res..

[14] Wen P.Y., Kesari S. (2008). Malignant gliomas in adults. N. Engl. J. Med..

[15] Czarnecka A.M., Campanella C., Zummo G., Cappello F. (2006). Mitochondrial chaperones in cancer: From molecular biology to clinical diagnostics. Cancer Biol. Ther..

[16] Macario A.J.L., Conway de Macario E. (2005). Sick chaperones, cellular stress, and disease. N. Engl. J. Med..

[17] Macario A.J.L., Conway de Macario E. (2007). Chaperonopathies and chaperonotherapy. FEBS Lett..

[18] Macario A.J.L., Cappello F., Zummo G., Conway de Macario E. (2010). Chaperonopathies of senescence and the scrambling of interactions between the chaperoning and the immune systems. Ann. N. Y. Acad. Sci..

[19] Macario A.J.L., Conway de Macario E., Cappello F. (2013). The Chaperonopathies: Diseases with Defective Molecular Chaperones.

[20] Macario A.J.L., Conway de Macario E., Fink G. Chaperone proteins and chaperonopathies. Stress Physiology, Biochemistry, and Pathology. Handbook of Stress.

[21] Lindquist S. (1986). The Heat-Shock Response. Annu. Rev. Biochem..

[22] Cappello F., Marino Gammazza A., Palumbo Piccionello A., Campanella C., Pace A., Conway de Macario E., Macario A.J.L. (2014). Hsp60 chaperonopathies and chaperonotherapy: Targets and agents. Expert Opin. Ther. Targets.

[23] Calderwood S.K., Stevenson M.A., Murshid A. (2012). Heat Shock Proteins, Autoimmunity, and Cancer Treatment. Autoimmune Dis..

[24] Tamura Y., Torigoe T., Kukita K., Saito K., Okuya K., Kutomi G., Hirata K., Sato N. (2012). Heat-shock proteins as endogenous ligands building a bridge between innate and adaptive immunity. Immunotherapy.

[25] Fagone P., Di Rosa M., Palumbo M., De Gregorio C., Nicoletti F., Malaguarnera L. (2012). Modulation of heat shock proteins during macrophage differentiation. Inflamm. Res..

[26] Rappa F., Farina F., Zummo G., David S., Campanella C., Carini F., Tomasello G., Damiani P., Cappello F., Conway de Macario E. (2012). Hsp-molecular chaperones in cancer biogenesis and tumor therapy: An overview. Anticancer Res..

[27] Kast R.E., Boockvar J.A., Brüning A., Cappello F., Chang W.-W., Cvek B., Dou Q.P., Duenas-Gonzalez A., Efferth T., Focosi D. (2013). A conceptually new treatment approach for relapsed glioblastoma: Coordinated undermining of survival paths with nine repurposed drugs (CUSP9) by the International Initiative for Accelerated Improvement of Glioblastoma Care. Oncotarget.

[28] Mattick J.S. (2003). Challenging the dogma: The hidden layer of non-protein-coding RNAs in complex organisms. Bioessays.

[29] Rodriguez A., Griffiths-Jones S., Ashurst J.L., Bradley A. (2004). Identification of mammalian microRNA host genes and transcription units. Genome Res..

[30] Batista P.J., Chang H.Y. (2013). Long noncoding RNAs: Cellular address codes in development and disease. Cell.

[31] Crea F., Clermont P.L., Parolia A., Wang Y., Helgason C.D. (2014). The non-coding transcriptome as a dynamic regulator of cancer metastasis. Cancer Metastasis Rev..

[32] Malone C.D., Hannon G.J. (2009). Small RNAs as guardians of the genome. Cell.

[33] Patil V.S., Zhou R., Rana T.M. (2014). Gene regulation by non-coding RNAs. Crit. Rev. Biochem. Mol. Biol..

[34] Brown B.D., Naldini L. (2009). Exploiting and antagonizing microRNA regulation for therapeutic and experimental applications. Nat. Rev. Genet..

[35] Huang W. (2017). MicroRNAs: Biomarkers, Diagnostics, and Therapeutics. Bioinform. MicroRNA Res..

[36] Borchert G.M., Lanier W., Davidson B.L. (2006). RNA polymerase III transcribes human microRNAs. Nat. Struct. Mol. Biol..

[37] Calin G.A., Dumitru C.D., Shimizu M., Bichi R., Zupo S., Noch E., Aldler H., Rattan S., Keating M., Rai K. (2002). Frequent deletions and down-regulation of micro-RNA genes miR15 and miR16 at 13q14 in chronic lymphocytic leukemia. Proc. Natl. Acad. Sci. USA.

[38] Calin G.A., Sevignani C., Dumitru C.D., Hyslop T., Noch E., Yendamuri S., Shimizu M., Rattan S., Bullrich F., Negrini M. (2004). Human microRNA genes are frequently located at fragile sites and genomic regions involved in cancers. Proc. Natl. Acad. Sci. USA.

[39] Zhang L., Huang J., Yang N., Greshock J., Megraw M.S., Giannakakis A., Liang S., Naylor T.L., Barchetti A., Ward M.R. (2006). microRNAs exhibit high frequency genomic alterations in human cancer. Proc. Natl. Acad. Sci. USA.

[40] Weber B., Stresemann C., Brueckner B., Lyko F. (2007). Methylation of Human MicroRNA Genes in Normal and Neoplastic Cells. Cell Cycle.

[41] Wang B., Hsu S., Wang X., Kutay H., Bid H.K., Yu J., Ganju R.K., Jacob S.T., Yuneva M., Ghoshal K. (2014). Reciprocal regulation of microRNA-122 and c-Myc in hepatocellular cancer: Role of E2F1 and transcription factor dimerization partner 2. Hepatology.

[42] Donzelli S., Mori F., Bellissimo T., Sacconi A., Casini B., Frixa T., Roscilli G., Aurisicchio L., Facciolo F., Pompili A. (2015). Epigenetic silencing of miR-145-5p contributes to brain metastasis. Oncotarget.

[43] Walz A.L., Ooms A., Gadd S., Gerhard D.S., Smith M.A., Guidry Auvil J.M., Meerzaman D., Chen Q.R., Hsu C.H., Yan C. (2015). Recurrent DGCR8, DROSHA, and SIX homeodomain mutations in favorable histology Wilms tumors. Cancer Cell.

[44] Ma L., Teruya-Feldstein J., Weinberg R.A. (2007). Tumour invasion and metastasis initiated by microRNA-10b in breast cancer. Nature.

[45] Li N., Fu H., Tie Y., Hu Z., Kong W., Wu Y., Zheng X. (2009). miR-34a inhibits migration and invasion by down-regulation of c-Met expression in human hepatocellular carcinoma cells. Cancer Lett..

[46] Volinia S., Calin G.A., Liu C.G., Ambs S., Cimmino A., Petrocca F., Visone R., Iorio M., Roldo C., Ferracin M. (2006). A microRNA expression signature of human solid tumors defines cancer gene targets. Proc. Natl. Acad. Sci. USA.

[47] Lee Y.S., Pressman S., Andress A.P., Kim K., White J.L., Cassidy J.J., Li X., Lubell K., Lim D.H., Cho I.S. (2009). Silencing by small RNAs is linked to endosomal trafficking. Nat. Cell Biol..

[48] Stalder L., Heusermann W., Sokol L., Trojer D., Wirz J., Hean J., Fritzsche A., Aeschimann F., Pfanzagl V., Basselet P. (2013). The rough endoplasmatic reticulum is a central nucleation site of siRNA-mediated RNA silencing. EMBO J..

[49] Barman B., Bhattacharyya S.N. (2015). mRNA Targeting to Endoplasmic Reticulum Precedes Ago Protein Interaction and MicroRNA (miRNA)-mediated Translation Repression in Mammalian Cells. J. Biol. Chem..

[50] Gibbings D.J., Ciaudo C., Erhardt M., Voinnet O. (2009). Multivesicular bodies associate with components of miRNA effector complexes and modulate miRNA activity. Nat. Cell Biol..

[51] Janas T., Janas M.M., Sapoń K., Janas T. (2015). Mechanisms of RNA loading into exosomes. FEBS Lett..

[52] Tewari M. (2015). A functional extracellular transcriptome in animals? Implications for biology, disease and medicine. Genome Biol..

[53] Squadrito M.L., Baer C., Burdet F., Maderna C., Gilfillan G.D., Lyle R., Ibberson M., De Palma M. (2014). Endogenous RNAs modulate microRNA sorting to exosomes and transfer to acceptor cells. Cell Rep..

[54] Neviani P., Fabbri M. (2015). Exosomic microRNAs in the Tumor Microenvironment. Front. Med..

[55] Caruso Bavisotto C., Marino Gammazza A., Rappa F., Fucarino A., Pitruzzella A., David S., Campanella C. (2013). Exosomes: Can doctors still ignore their existence?. EuroMediterr. Biomed. J..

[56] Vlassov A.V., Magdaleno S., Setterquist R., Conrad R. (2012). Exosomes: Current knowledge of their composition, biological functions, and diagnostic and therapeutic potentials. Biochim. Biophys. Acta-Gen. Subj..

[57] Pan B.T., Blostein R., Johnstone R.M. (1983). Loss of the transferrin receptor during the maturation of sheep reticulocytes in vitro. An immunological approach. Biochem. J..

[58] Logozzi M., De Milito A., Lugini L., Borghi M., Calabrò L., Spada M., Perdicchio M., Marino M.L., Federici C., Iessi E. (2009). High Levels of Exosomes Expressing CD63 and Caveolin-1 in Plasma of Melanoma Patients. PLoS ONE.

[59] Cappello F., Logozzi M., Campanella C., Caruso Bavisotto C., Marcilla A., Properzi F., Fais S. (2017). Exosome levels in human body fluids: A tumor marker by themselves?. Eur. J. Pharm. Sci..

[60] Raposo G., Stoorvogel W. (2013). Extracellular vesicles: Exosomes, microvesicles, and friends. J. Cell Biol..

[61] Van Niel G., Porto-Carreiro I., Simoes S., Raposo G. (2006). Exosomes: A common pathway for a specialized function. J. Biochem..

[62] Ostrowski M., Carmo N.B., Krumeich S., Fanget I., Raposo G., Savina A., Moita C.F., Schauer K., Hume A.N., Freitas R.P. (2010). Rab27a and Rab27b control different steps of the exosome secretion pathway. Nat. Cell Biol..

[63] Xie Y., Zhang H., Li W., Deng Y., Munegowda M.A., Chibbar R., Qureshi M., Xiang J. (2010). Dendritic cells recruit T cell exosomes via exosomal LFA-1 leading to inhibition of CD8+ CTL responses through downregulation of peptide/MHC class I and Fas ligand-mediated cytotoxicity. J. Immunol..

[64] Van Niel G., Charrin S., Simoes S., Romao M., Rochin L., Saftig P., Marks M.S., Rubinstein E., Raposo G. (2011). The tetraspanin CD63 regulates ESCRT-independent and -dependent endosomal sorting during melanogenesis. Dev. Cell.

[65] Schorey J.S., Bhatnagar S. (2008). Exosome function: From tumor immunology to pathogen biology. Traffic.

[66] Choi D.-S., Lee J.-M., Park G.W., Lim H.-W., Bang J.Y., Kim Y.-K., Kwon K.-H., Kwon H.J., Kim K.P., Gho Y.S. (2007). Proteomic analysis of microvesicles derived from human colorectal cancer cells. J. Proteome Res..

[67] Qu Y., Franchi L., Nunez G., Dubyak G.R. (2007). Nonclassical IL-1 β secretion stimulated by P2X7 receptors is dependent on inflammasome activation and correlated with exosome release in murine macrophages. J. Immunol..

[68] Phoonsawat W., Aoki-Yoshida A., Tsuruta T., Sonoyama K. (2014). Adiponectin is partially associated with exosomes in mouse serum. Biochem. Biophys. Res. Commun..

[69] Chen L., Chen R., Kemper S., Charrier A., Brigstock D.R. (2015). Suppression of fibrogenic signaling in hepatic stellate cells by Twist1-dependent microRNA-214 expression: Role of exosomes in horizontal transfer of Twist1. Am. J. Physiol. Gastrointest. Liver Physiol..

[70] Campanella C., Bucchieri F., Merendino A.M., Fucarino A., Burgio G., Corona D.F.V., Barbieri G., David S., Farina F., Zummo G. (2012). The odyssey of Hsp60 from tumor cells to other destinations includes plasma membrane-associated stages and Golgi and exosomal protein-trafficking modalities. PLoS ONE.

[71] Campanella C., Rappa F., Sciumè C., Marino Gammazza A., Barone R., Bucchieri F., David S., Curcurù G., Caruso Bavisotto C., Pitruzzella A. (2015). Heat shock protein 60 levels in tissue and circulating exosomes in human large bowel cancer before and after ablative surgery. Cancer.

[72] Lancaster G.I., Febbraio M.A. (2005). Exosome-dependent trafficking of Hsp70: A novel secretory pathway for cellular stress proteins. J. Biol. Chem..

[73] Bausero M.A., Gastpar R., Multhoff G., Asea A. (2005). Alternative mechanism by which IFN-gamma enhances tumor recognition: Active release of heat shock protein 72. J. Immunol..

[74] Vega V.L., Rodríguez-Silva M., Frey T., Gehrmann M., Diaz J.C., Steinem C., Multhoff G., Arispe N., De Maio A. (2008). Hsp70 translocates into the plasma membrane after stress and is released into the extracellular environment in a membrane-associated form that activates macrophages. J. Immunol..

[75] Gastpar R., Gehrmann M., Bausero M.A., Asea A., Gross C., Schroeder J.A., Multhoff G. (2005). Heat shock protein 70 surface-positive tumor exosomes stimulate migratory and cytolytic activity of natural killer cells. Cancer Res..

[76] Lv L.-H., Wan Y.-L., Lin Y., Zhang W., Yang M., Li G.-L., Lin H.-M., Shang C.-Z., Chen Y.-J., Min J. (2012). Anticancer drugs cause release of exosomes with heat shock proteins from human hepatocellular carcinoma cells that elicit effective natural killer cell antitumor responses in vitro. J. Biol. Chem..

[77] McCready J., Sims J.D., Chan D., Jay D.G. (2010). Secretion of extracellular Hsp90α via exosomes increases cancer cell motility: A role for plasminogen activation. BMC Cancer.

[78] Merendino A.M., Bucchieri F., Campanella C., Marcianò V., Ribbene A., David S., Zummo G., Burgio G., Corona D.F.V., Conway de Macario E. (2010). Hsp60 is actively secreted by human tumor cells. PLoS ONE.

[79] Gupta S., Knowlton A.-A. (2007). Hsp60 trafficking in adult cardiac myocytes: Role of the exosomal pathway. Am. J. Physiol. Heart Circ. Physiol..

[80] Hayoun D., Kapp T., Edri-Brami M., Ventura T., Cohen M., Avidan A., Lichtenstein R.G. (2012). Hsp60 is transported through the secretory pathway of 3-MCA-induced fibrosarcoma tumour cells and undergoes N-glycosylation. FEBS J..

[81] Marino Gammazza A., Campanella C., Barone R., Caruso Bavisotto C., Gorska M., Wozniak M., Carini F., Cappello F., D’Anneo A., Lauricella M. (2017). Doxorubicin anti-tumor mechanisms include Hsp60 post-translational modifications leading to the Hsp60/p53 complex dissociation and instauration of replicative senescence. Cancer Lett..

[82] Ratajczak J., Miekus K., Kucia M., Zhang J., Reca R., Dvorak P., Ratajczak M.Z. (2006). Embryonic stem cell-derived microvesicles reprogram hematopoietic progenitors: Evidence for horizontal transfer of mRNA and protein delivery. Leukemia.

[83] Kosaka N., Iguchi H., Yoshioka Y., Takeshita F., Matsuki Y., Ochiya T. (2010). Secretory mechanisms and intercellular transfer of microRNAs in living cells. J. Biol. Chem..

[84] Villarroya-Beltri C., Gutiérrez-Vázquez C., Sánchez-Cabo F., Pérez-Hernández D., Vázquez J., Martin-Cofreces N., Martinez-Herrera D.J., Pascual-Montano A., Mittelbrunn M., Sánchez-Madrid F. (2013). Sumoylated hnRNPA2B1 controls the sorting of miRNAs into exosomes through binding to specific motifs. Nat. Commun..

[85] Guduric-Fuchs J., O’Connor A., Camp B., O’Neill C.L., Medina R.J., Simpson D.A. (2012). Selective extracellular vesicle-mediated export of an overlapping set of microRNAs from multiple cell types. BMC Genom..

[86] Nolte’T Hoen E.N.M., Buermans H.P.J., Waasdorp M., Stoorvogel W., Wauben M.H.M., ’T Hoen P.A.C. (2012). Deep sequencing of RNA from immune cell-derived vesicles uncovers the selective incorporation of small non-coding RNA biotypes with potential regulatory functions. Nucleic Acids Res..

[87] Li C.C.Y., Eaton S.A., Young P.E., Lee M., Shuttleworth R., Humphreys D.T., Grau G.E., Combes V., Bebawy M., Gong J. (2013). Glioma microvesicles carry selectively packaged coding and non-coding RNAs which alter gene expression in recipient cells. RNA Biol..

[88] Schiera G., Di Liegro C.M., Di Liegro I. (2017). Molecular Determinants of Malignant Brain Cancers: From Intracellular Alterations to Invasion Mediated by Extracellular Vesicles. Int. J. Mol. Sci..

[89] Ngolab J., Trinh I., Rockenstein E., Mante M., Florio J., Trejo M., Masliah D., Adame A., Masliah E., Rissman R.A. (2017). Brain-derived exosomes from dementia with Lewy bodies propagate α-synuclein pathology. Acta Neuropathol. Commun..

[90] Picciolini S., Gualerzi A., Vanna R., Sguassero A., Gramatica F., Bedoni M., Masserini M., Morasso C. (2018). Detection and Characterization of Different Brain-Derived Subpopulations of Plasma Exosomes by Surface Plasmon Resonance Imaging. Anal. Chem..

[91] Karnati H.K., Garcia J.H., Tweedie D., Becker R.E., Kapogiannis D., Greig N.H. (2018). Neuronal Enriched Extracellular Vesicle Proteins as Biomarkers for Brain Traumatic Injury. J. Neurotrauma.

[92] Mrowczynski O.D., Zacharia B.E., Connor J.R. (2018). Exosomes and their implications in central nervous system tumor biology. Prog. Neurobiol..

[93] Chen C.C., Liu L., Ma F., Wong C.W., Guo X.E., Chacko J.V., Farhoodi H.P., Zhang S.X., Zimak J., Ségaliny A. (2016). Elucidation of Exosome Migration Across the Blood–Brain Barrier Model In Vitro. Cell. Mol. Bioeng..

[94] Yuan D., Zhao Y., Banks W.A., Bullock K.M., Haney M., Batrakova E., Kabanov A.V. (2017). Macrophage exosomes as natural nanocarriers for protein delivery to inflamed brain. Biomaterials.

[95] Graner M., Redzic J., Ung T. (2014). Glioblastoma extracellular vesicles: Reservoirs of potential biomarkers. Pharmgenom. Pers. Med..

[96] Chun S., Ahn S., Yeom C.-H., Park S. (2016). Exosome Proteome of U-87MG Glioblastoma Cells. Biology.

[97] Mallawaaratchy D.M., Hallal S., Russell B., Ly L., Ebrahimkhani S., Wei H., Christopherson R.I., Buckland M.E., Kaufman K.L. (2017). Comprehensive proteome profiling of glioblastoma-derived extracellular vesicles identifies markers for more aggressive disease. J. Neurooncol..

[98] Skog J., Würdinger T., van Rijn S., Meijer D.H., Gainche L., Sena-Esteves M., Curry W. T., Carter B.S., Krichevsky A.M., Breakefield X.O. (2008). Glioblastoma microvesicles transport RNA and proteins that promote tumour growth and provide diagnostic biomarkers. Nat. Cell Biol..

[99] Yoshimoto K., Dang J., Zhu S., Nathanson D., Huang T., Dumont R., Seligson D.B., Yong W.H., Xiong Z., Rao N. (2008). Development of a real-time RT-PCR assay for detecting EGFRvIII in glioblastoma samples. Clin. Cancer Res..

[100] Strik H.M., Weller M., Frank B., Hermisson M., Deininger M.H., Dichgans J., Meyermann R. (2000). Heat shock protein expression in human gliomas. Anticancer Res..

[101] Shen G., Liang S., Xu Z., Zhou L., Xiao S., Xia X., Li R., Liao Y., You C., Wei Y. (2010). Downregulated expression of Hsp27 in human low-grade glioma tissues discovered by a quantitative proteomic analysis. Proteome Sci..

[102] Khalil A.A., Kabapy N.F., Deraz S.F., Smith C. (2011). Heat shock proteins in oncology: Diagnostic biomarkers or therapeutic targets?. Biochim. Biophys. Acta.

[103] Golembieski W.A., Thomas S.L., Schultz C.R., Yunker C.K., McClung H.M., Lemke N., Cazacu S., Barker T., Sage E.H., Brodie C. (2008). Hsp27 mediates SPARC-induced changes in glioma morphology, migration, and invasion. Glia.

[104] Li G., Xu Y., Guan D., Liu Z., Liu D.X. (2011). Hsp70 protein promotes survival of C6 and U87 glioma cells by inhibition of ATF5 degradation. J. Biol. Chem..

[105] Sauvageot C.M.-E., Weatherbee J.L., Kesari S., Winters S.E., Barnes J., Dellagatta J., Ramakrishna N.R., Stiles C.D., Kung A.L.-J., Kieran M.W. (2009). Efficacy of the Hsp90 inhibitor 17-AAG in human glioma cell lines and tumorigenic glioma stem cells. Neuro. Oncol..

[106] Nagaraju G.P., Long T.-E., Park W., Landry J.C., Taliaferro-Smith L., Farris A.B., Diaz R., El-Rayes B.F. (2015). Heat shock protein 90 promotes epithelial to mesenchymal transition, invasion, and migration in colorectal cancer. Mol. Carcinog..

[107] Rappa F., Cappello F., Halatsch M.-E., Scheuerle A., Kast R.E. (2013). Aldehyde dehydrogenase and Hsp90 co-localize in human glioblastoma biopsy cells. Biochimie.

[108] Rappa F., Unti E., Baiamonte P., Cappello F., Scibetta N. (2013). Different immunohistochemical levels of Hsp60 and Hsp70 in a subset of brain tumors and putative role of Hsp60 in neuroepithelial tumorigenesis. Eur. J. Histochem..

[109] Clayton A., Turkes A., Navabi H., Mason M.D., Tabi Z. (2005). Induction of heat shock proteins in B-cell exosomes. J. Cell Sci..

[110] Campanella C., Caruso Bavisotto C., Marino Gammazza A., Nikolic D., Rappa F., David S., Cappello F., Bucchieri F., Fais S. (2014). Exosomal Heat Shock Proteins as New Players in Tumour Cell-to-cell Communication. J. Circ. Biomark..

[111] Gimenez M., Marie S.K.N., Oba-Shinjo S., Uno M., Izumi C., Oliveira J.B., Rosa J.C. (2015). Quantitative proteomic analysis shows differentially expressed HspB1 in glioblastoma as a discriminating short from long survival factor and NOVA1 as a differentiation factor between low-grade astrocytoma and oligodendroglioma. BMC Cancer.

[112] Thuringer D., Hammann A., Benikhlef N., Fourmaux E., Bouchot A., Wettstein G., Solary E., Garrido C. (2011). Transactivation of the epidermal growth factor receptor by heat shock protein 90 via Toll-like receptor 4 contributes to the migration of glioblastoma cells. J. Biol. Chem..

[113] Cheng W., Li Y., Hou X., Zhang N., Ma J., Ding F., Li F., Miao Z., Zhang Y., Qi Q. (2014). Hsp60 is involved in the neuroprotective effects of naloxone. Mol. Med. Rep..

[114] Mäkelä K.S., Haapasalo J.A., Ilvesaro J.M., Parkkila S., Paavonen T., Haapasalo H.K. (2014). Hsp27 and its expression pattern in diffusely infiltrating astrocytomas. Histol. Histopathol..

[115] Graner M.W., Raynes D.A., Bigner D.D., Guerriero V. (2009). Heat shock protein 70-binding protein 1 is highly expressed in high-grade gliomas, interacts with multiple heat shock protein 70 family members, and specifically binds brain tumor cell surfaces. Cancer Sci..

[116] Morino M., Tsuzuki T., Ishikawa Y., Shirakami T., Yoshimura M., Kiyosuke Y., Matsunaga K., Yoshikumi C., Saijo N. (1997). Specific expression of Hsp27 in human tumor cell lines in vitro. In Vivo.

[117] Scott K.A., Dennis J.L., Dalgleish A.G., Liu W.M. (2015). Inhibiting Heat Shock Proteins Can Potentiate the Cytotoxic Effect of Cannabidiol in Human Glioma Cells. Anticancer Res..

[118] Ghosh J.C., Siegelin M.D., Dohi T., Altieri D.C. (2010). Heat shock protein 60 regulation of the mitochondrial permeability transition pore in tumor cells. Cancer Res..

[119] Alexiou G.A., Vartholomatos G., Stefanaki K., Patereli A., Dova L., Karamoutsios A., Lallas G., Sfakianos G., Moschovi M., Prodromou N. (2013). Expression of heat shock proteins in medulloblastoma. J. Neurosurg. Pediatr..

[120] Marino Gammazza A., Caruso Bavisotto C., David S., Barone R., Rappa F., Campanella C., Conway de Macario E., Cappello F., Macario A.J.L. (2017). Hsp60 is a ubiquitous player in the physiological and pathogenic interactions between the chaperoning and the immune systems. Curr. Immunol. Rev..

[121] Samali A., Cai J., Zhivotovsky B., Jones D.P., Orrenius S. (1999). Presence of a pre-apoptotic complex of pro-caspase-3, Hsp60 and Hsp10 in the mitochondrial fraction of jurkat cells. EMBO J..

[122] Caruso Bavisotto C., Nikolic D., Marino Gammazza A., Barone R., Lo Cascio F., Mocciaro E., Zummo G., Conway de Macario E., Macario A.J.L., Cappello F. (2017). The dissociation of the Hsp60/pro-Caspase-3 complex by bis(pyridyl)oxadiazole copper complex (CubipyOXA) leads to cell death in NCI-H292 cancer cells. J. Inorg. Biochem..

[123] Kirchhoff S.R., Gupta S., Knowlton A.A. (2002). Cytosolic heat shock protein 60, apoptosis, and myocardial injury. Circulation.

[124] Stefano L., Racchetti G., Bianco F., Passini N., Gupta R.S., Panina Bordignon P., Meldolesi J. (2009). The surface-exposed chaperone, Hsp60, is an agonist of the microglial TREM2 receptor. J. Neurochem..

[125] Creagh E.M., Carmody R.J., Cotter T.G. (2000). Heat shock protein 70 inhibits caspase-dependent and -independent apoptosis in Jurkat T cells. Exp. Cell Res..

[126] Belkacemi L., Hebb M.O. (2014). Hsp27 knockdown produces synergistic induction of apoptosis by Hsp90 and kinase inhibitors in glioblastoma multiforme. Anticancer Res..

[127] Wu Z.B., Cai L., Lin S.J., Leng Z.G., Guo Y.H., Yang W.L., Chu Y.W., Yang S.-H., Zhao W.G. (2016). Heat Shock Protein 47 Promotes Glioma Angiogenesis. Brain Pathol..

[128] Trentin G.A., He Y., Wu D.C., Tang D., Rozakis-Adcock M. (2004). Identification of a hTid-1 mutation which sensitizes gliomas to apoptosis. FEBS Lett..

[129] Itoh H., Komatsuda A., Wakui H., Miura A.B., Tashima Y. (1999). Mammalian Hsp60 is a major target for an immunosuppressant mizoribine. J. Biol. Chem..

[130] Wadhwa R., Sugihara T., Yoshida A., Nomura H., Reddel R.R., Simpson R., Maruta H., Kaul S.C. (2000). Selective toxicity of MKT-077 to cancer cells is mediated by its binding to the hsp70 family protein mot-2 and reactivation of p53 function. Cancer Res..

[131] Jego G., Hazoumé A., Seigneuric R., Garrido C. (2013). Targeting heat shock proteins in cancer. Cancer Lett..

[132] Lamberti D., Cristinziano G., Porru M., Leonetti C., Egan J.B., Shi C.-X., Buglioni S., Amoreo C.A., Castellani L., Borad M.J. (2018). Hsp90 inhibition drives degradation of FGFR2 fusion proteins: Implications for treatment of cholangiocarcinoma. Hepatology.

[133] Pan J., Jiang F., Zhou J., Wu D., Sheng Z., Li M. (2018). Hsp90: A Novel Target Gene of miRNA-628-3p in A549 Cells. Biomed Res. Int..

[134] Li G., Cai M., Fu D., Chen K., Sun M., Cai Z., Cheng B. (2012). Heat shock protein 90B1 plays an oncogenic role and is a target of microRNA-223 in human osteosarcoma. Cell. Physiol. Biochem..

[135] Kariya A., Furusawa Y., Yunoki T., Kondo T., Tabuchi Y. (2014). A microRNA-27a mimic sensitizes human oral squamous cell carcinoma HSC-4 cells to hyperthermia through downregulation of Hsp110 and Hsp90. Int. J. Mol. Med..

[136] MacKenzie T.N., Mujumdar N., Banerjee S., Sangwan V., Sarver A., Vickers S., Subramanian S., Saluja A.K. (2013). Triptolide induces the expression of miR-142-3p: A negative regulator of heat shock protein 70 and pancreatic cancer cell proliferation. Mol. Cancer Ther..

[137] Shan Z.-X., Lin Q.-X., Deng C.-Y., Zhu J.-N., Mai L.-P., Liu J.-L., Fu Y.-H., Liu X.-Y., Li Y.-X., Zhang Y.-Y. (2010). miR-1/miR-206 regulate Hsp60 expression contributing to glucose-mediated apoptosis in cardiomyocytes. FEBS Lett..

[138] Fang Y., Xie T., Xue N., Kuang Q., Wei Z., Liang M., Ding X. (2017). miR-382 Contributes to Renal Tubulointerstitial Fibrosis by Downregulating HSPD1. Oxid. Med. Cell. Longev..

[139] Choghaei E., Khamisipour G., Falahati M., Naeimi B., Mossahebi-Mohammadi M., Tahmasebi R., Hasanpour M., Shamsian S., Hashemi Z.S. (2016). Knockdown of microRNA-29a Changes the Expression of Heat Shock Proteins in Breast Carcinoma MCF-7 Cells. Oncol. Res. Featur. Preclin. Clin. Cancer Ther..

[140] Barbagallo G.M.V, Paratore S., Caltabiano R., Palmucci S., Parra H.S., Privitera G., Motta F., Lanzafame S., Scaglione G., Longo A. (2014). Long-term therapy with temozolomide is a feasible option for newly diagnosed glioblastoma: A single-institution experience with as many as 101 temozolomide cycles. Neurosurg. Focus.

[141] Maugeri R., Villa A., Pino M., Imperato A., Giammalva G.R., Costantino G., Graziano F., Gulì C., Meli F., Francaviglia N. (2018). With a little help from my friends: The role of intraoperative fluorescent dyes in the surgical management of high-grade gliomas. Brain Sci..

[142] Zhang L., Wang S., Wangtao, Wang Y., Wang J., Jiang L., Li S., Hu X., Wang Q. (2009). Upregulation of GRP78 and GRP94 and its function in chemotherapy resistance to VP-16 in human lung cancer cell line SK-MES-1. Cancer Investig..

[143] Zhang X., Zhang L., Wang S., Wu D., Yang W. (2015). Decreased functional expression of Grp78 and Grp94 inhibits proliferation and attenuates apoptosis in a human gastric cancer cell line in vitro. Oncol. Lett..

[144] Masui K., Cloughesy T.F., Mischel P.S. (2012). Molecular pathology in adult high-grade gliomas: From molecular diagnostics to target therapies. Neuropathol. Appl. Neurobiol..

[145] Xu C., Lu Y., Pan Z., Chu W., Luo X., Lin H., Xiao J., Shan H., Wang Z., Yang B. (2007). The muscle-specific microRNAs miR-1 and miR-133 produce opposing effects on apoptosis by targeting Hsp60, Hsp70 and caspase-9 in cardiomyocytes. J. Cell Sci..

[146] Pan Z., Sun X., Ren J., Li X., Gao X., Lu C., Zhang Y., Sun H., Wang Y., Wang H. (2012). miR-1 Exacerbates Cardiac Ischemia-Reperfusion Injury in Mouse Models. PLoS ONE.

[147] Neumann E., Brandenburger T., Santana-Varela S., Deenen R., Köhrer K., Bauer I., Hermanns H., Wood J.N., Zhao J., Werdehausen R. (2016). MicroRNA-1-associated effects of neuron-specific brain-derived neurotrophic factor gene deletion in dorsal root ganglia. Mol. Cell. Neurosci..

[148] Evert B.O., Nalavade R., Jungverdorben J., Matthes F., Weber S., Rajput A., Bonn S., Brüstle O., Peitz M., Krauß S. (2018). Upregulation of miR-370 and miR-543 is associated with reduced expression of heat shock protein 40 in spinocerebellar ataxia type 3. PLoS ONE.

[149] Graziano F., Caruso Bavisotto C., Marino Gammazza A., Rappa F., Conway de Macario E., Macario A.J.L., Cappello F., Campanella C., Maugeri R., Iacopino D.G. (2018). Chaperonology: The third eye on brain gliomas. Brain Sci..

